# Nested Binary Classifier as an Outlier Detection Method in Human Activity Recognition Systems

**DOI:** 10.3390/e25081121

**Published:** 2023-07-26

**Authors:** Agnieszka Duraj, Daniel Duczymiński

**Affiliations:** Institute of Information Technology, Lodz University of Technology, al. Politechniki 8, 93-590 Łódź, Poland

**Keywords:** outliers, anomalies, human activity recognition, machine learning, classification, deep learning

## Abstract

The present article is devoted to outlier detection in phases of human movement. The aim was to find the most efficient machine learning method to detect abnormal segments inside physical activities in which there is a probability of origin from other activities. The problem was reduced to a classification task. The new method is proposed based on a nested binary classifier. Test experiments were then conducted using several of the most popular machine learning algorithms (linear regression, support vector machine, *k*-nearest neighbor, decision trees). Each method was separately tested on three datasets varying in characteristics and number of records. We set out to evaluate the effectiveness of the models, basic measures of classifier evaluation, and confusion matrices. The nested binary classifier was compared with deep neural networks. Our research shows that the method of nested binary classifiers can be considered an effective way of recognizing outlier patterns for HAR systems.

## 1. Introduction

Daily processing of huge databases (BIG DATA) leads to the development of decision support systems and expert systems, but above all, to the development of intelligent data processing algorithms. These algorithms facilitate the data mining process on every level. It is extremely important to detect outliers in this aspect because an object detected as an outlier can affect the further process of intelligent data analysis. It can lead to errors in classification or grouping, or it can be a discovery of a new phenomenon, gene, disease, or an object with hitherto unknown characteristics.

In scientific articles, one can find a rich variety of outlier definitions, as well as a variety of methods presented. The outlier will be defined differently in applications related to identifying the characteristics of satellite images, and differently in detecting congestion or intrusions into computer networks. Another definition of an outlier can be found in machine diagnostics or for the identification of new molecular structures in pharmaceutical research as well as medical diagnostics. In the above cases, the outlier is treated as a result of a damaged machine, a measurement error, or it is a kind of distinctiveness, e.g., it has other features or other attribute values unknown so far.

Anomaly detection is also a very important element of machine learning. It is the process of identifying unusual deviations in large datasets, i.e., observations that do not fit a particular pattern. By distorting the quality of data, they can have a negative impact on the correct interpretation of statistics and the course of learning. Depending on the scale of the problem, they can prolong the training of models and even significantly reduce the effectiveness of estimation, prediction, or classification.

The rapid development of new technologies applied in everyday activities, or the ability to record data from various sensors and devices, leads to the automation of not only the process of collecting and storing the data, but also the development of data mining and machine learning algorithms. A quick analysis of data from various sensors that monitor factors like everyday human activities enables productive planning of learning, training, diets, and monitoring of human activity or health.

Sensors in wristbands, smartwatches, or mobile phones can have many applications in routine medical and everyday activities, such as gait analysis, healthcare, fitness, etc. In this article, the accelerometer and gyroscope sensor dataset has been used to propose an effective model of physical activity detection. Human activity recognition (HAR) is a research area dealing with the classification or prediction of activities performed by a specific person. In HAR systems, data are collected from a gyroscope or accelerometer in a smartphone. Activities such as sitting, walking, climbing stairs, going downstairs, standing, and lying down are recognized. Detection of activity and phases of human movements has many applications, especially in monitoring systems, security, smart homes, and health care.

This problem is very important, especially among elderly or disabled people. It is often necessary to monitor the patient after various surgical procedures while performing daily, routine activities at home or away from home.

To recognize human activity, machine learning algorithms, such as the k-nearest neighbors, random forest, and support vector machine, are used, but also deep learning algorithms, such as artificial neural networks and long short-term memory networks.

In the literature, according to the authors’ knowledge, there are no papers dealing with the problem of detecting outliers in HAR systems. The research problem considered in this article is therefore very important and up to date. This article focuses on the detection of anomalous segments representing human physical activities. Outliers, in this case, are fragments produced by different, unusual phases of movement. We do not detect noise as outliers. In this paper, we detect outliers as unusual activities that are not yet known, labeled, or have appeared suddenly, e.g., they occur inside other activities.

This article presents the experiments carried out by the authors, as a result of which the effectiveness of selected machine learning algorithms in detecting outliers segments related to human activities was examined. The research was carried out on three numeric datasets. These sets were pre-processed and cleaned of foreign patterns, i.e., missing, erroneous, or empty records. These types of gaps or empty records are foreign patterns that can be treated as outliers that can be removed because they do not affect the analyzed data. In this article, the authors focus on detecting unusual activities and unexpected events that occur randomly. The detection of outlier patterns defined in this way is undoubtedly a novelty of the topic undertaken by the authors.

The present article has the following structure: Section 2 defines the human activities in HAR systems and outlier detection. Next, Section 3 defines the problem discussed in the paper. Outliers in the context of human movement phases are explained. The proposed nested algorithm is described in Section 4 and the research method in Section 5. The experiments and results are then listed under Section 6. The paper ends in a discussion in Section 7 and a conclusion in Section 8.

## 2. Related Work

In this article, we consider the outlier detection problem. We detect unusual outlier activity in HAR systems. This activity occurs very rarely. Many publications concern the classification of simple activity phases in HAR systems (such as, for example, walking and lying down). For this reason, this section has been divided into two parts. In Section 2.1 the work on the classification of human activity in HAR systems is reviewed. In Section 2.2 the most important works related to detecting exceptions are presented.

### 2.1. Classification of Human Activity Recognition

In the case of recognizing the activity of human movement phases, we are dealing with the task of classifying time series. The data subjected to classification are collected from specialized sensors, but also from everyday devices such as smartphones and smartwatches. In this case, pre-processing of the input data is assumed, a feature vector based on overlapping time series windows of a fixed size is created, and finally, classifier models are trained on the input data.

The detection of phases of human movements can be recognized by machine learning algorithms. A commonly used approach is to employ a support vector machine. For example, the authors in paper [1] showed that a multiclass SVM machine produces accurate results classifying human activity. The authors of [2] proposed the SVM algorithm for recognizing 12 human movements. The data were taken from the sensor (Microsoft Kinect) installed in the smart home system. The support vector machine in human motion classification tasks was also used in [3,4]. In turn, the works of [5,6,7] propose methods based on the k-nearest neighbors algorithm (k-NN). You can also find works presenting the use of other classification algorithms, such as the naive Bayes algorithm, decision trees, random forests, or hybrid methods combining several classification algorithms at various stages. See, for example, the combination of decision trees and the Bayes classifier in [8], or convolutional neural networks and the Bayesian classifier in [9]. In the work of [10], the authors used the k-nearest neighbors algorithm (k-NN) and a random forest to improve the efficiency of classification models in recognizing human activities. Multivariate outlier detection was used to improve the quality of the training data by removing outliers. In k-NN, the performance increased from 55.9% to 63.59%. The data consisted of readings from a wrist-mounted triaxial accelerometer, which were then compressed into overlapping time windows. From the obtained segments, 51 features were calculated, i.e., average angular velocity, average logarithmic velocity, maximum, minimum, standard deviation, etc. The examined activities include personal hygiene, food preparation, and sports activities. In recent years, convolutional neural networks and deep learning have been proposed in HAR systems for recognizing phases of human movement.

In [11], the authors presented a novel technique for detecting anomalous segments in raw time sequences. Outlier detection methods focus on finding anomalous data samples that were probably generated by another mechanism, but also on sub-actions inside the main activities. An algorithm for the automatic characterization of DRNN time series was proposed, which, in turn, inspired LSTM networks implemented to predict incoming time data segments.The proposed algorithm was verified by the authors based on data from sensors worn on the body (accelerometer). Data for four different activities (walking, going up the stairs, going down the stairs, and running) and two different datasets were used for training and testing.

Our proposed method was checked using three numeric datasets, and the outlier detection in images is omitted in this paper. This paper takes into account only the outlier activity classification problem. The works related to numerical data and classification methods have been included above. It should be emphasized that innovative works related to the subject of activity detection in HAR systems appear in the literature. For example, in [12], the authors proposed a simple but effective feature matching method to remove mismatches for remote sensing in images.

Other papers deal with clustering methods. For example, in [13], the authors proposed a hierarchical clustering (HC)-based outlier detection technique to remove the outliers. The IHPTDL-HAR technique incorporates a DL-based deep belief network. In [14], the authors presented an online continual learning (OCL) scenario for HAR. The authors described a technique, OCL-HAR, that generated real-time predictions based on the streaming sensor data while, at the same time, discovering and learning new activities.

### 2.2. Outlier Detection

In scientific articles, one can find a wide variety of outlier definitions, as well as a variety of presented methods for detecting them. The definition of the outlier is very intuitive, therefore, the outlier will be defined differently in applications related to identifying the characteristics of satellite images, and differently when detecting congestion or intrusions into computer networks. Yet another definition of the outlier can be found for machine diagnostics, or the identification of new molecular structures in pharmaceutical research as well as medical diagnostics. In medicine, outliers are often used to describe various types of disease anomalies, e.g., cardiac arrhythmias and epilepsy. In the above cases, the outlier is treated as the result of a damaged machine, a measurement error, or it is a kind of distinctiveness, e.g., it has other features or other attribute values unknown so far. Numerous examples can be given related to outlier detection in a given context, in a designated problem domain. For example:

- Outliers such as computer network hacking [15,16];
- Outliers such as fraudulent transactions on bank accounts or credit cards [17];
- Outliers such as illegal activities on customer accounts in CRM systems, e.g., call activity, phone text messages [18];
- Outliers such as manufacturing defects, assembly line defects [19];
- Outliers such as unusual public monitoring events [20];
- Outliers such as disease anomalies [21];
- Outliers such as genetic changes, new molecular structures [22].

Several publications on outlier detection from a specific application point of view are listed. It is easy to see from this that outliers have a distinct character. They operate in different spaces, they are a different type of data. Outlier detection methods must therefore be targeted not only to the context of the problem domain, but they also must adapt to the type of data (numeric, text, mixed, spatial, image, etc.). In this respect, outlier detection methods can be divided into statistical methods, distance-based methods, and density-based methods. We can also divide outlier detection methods according to the division of machine learning methods. Here, we distinguish four types: supervised learning, semi-supervised learning, unsupervised learning, and reinforcement learning.

## 3. Problem Statement

### 3.1. The Definition of Outlier

The definition of an outlier is intuitive, depending on the type of data or domain of the problem under consideration. According to the definition given in [23], an outlier is an observation that differs so much from the others that it raises suspicion that it was generated by another mechanism. However, according to [24], an outlier is an observation or a subset of observations that seem inconsistent with the rest of the dataset. In [25], it is stated that outliers are patterns in the data that do not conform to the well-defined notion of normal behavior.

It should therefore be assumed that an outlier is most often an undesirable deviation from the norm, something that may distort the entirety of datasets and, as a consequence, lead to, for example, incorrect values of statistical measures, such as the mean or standard deviation, and increase the data classification error. When it comes to machine learning algorithms, they are sensitive to the range and distribution of attribute values. The presence of outliers can therefore confuse the entire process, resulting in longer training times, less accurate models, and, ultimately, poorer results. Analyzing data containing outliers, without their detection, leads to distorted or completely incorrect inference results. Undoubtedly, the type of outliers one deals with depends mostly on the types of data being used. However, both in statistics and data science, it has been assumed that anomalies can be divided into three main categories. We distinguish:

- Global outliers (point anomalies)—an observation (data point) is considered an outlier if its value extends significantly beyond the entirety of the dataset in which it resides.
- Contextual outliers (conditional anomalies)—values are within the global range but are abnormal compared to the seasonal pattern. A seasonal pattern has a constant frequency in which the time series is influenced by seasonal factors, i.e., the season of the year or the day of the week.
- Collective outliers—a subset of data are considered anomalous if all of these values as a set deviate significantly from the whole. At the same time, its values are not anomalous in themselves either in a global or contextual sense.

### 3.2. Outliers in the Context of Human Movement Phases

Activity recognition is a bit different from a typical classification task because, in this case, a series of data points collected over a period of time is required as a training set. Most of the available scientific sources and articles devoted to this subject state that one of two types of datasets is used to train models that recognize activities depending on what is to be achieved. The first type is numerical data, i.e., the measurement results of inertial sensors placed on the test object. Some of the most popular may be the accelerometer and the gyroscope. They are embedded, for example, in most of today’s mobile phones. They allow the determination of linear and angular acceleration, as well as the orientation, of the device in three-dimensional space. Collecting a large number of samples and processing them appropriately can lay the foundations for a good learning kit for machine learning methods. Activity monitoring with accelerometer readings most often concerns three phases: standing, lying down, and walking. The second type of datasets contain image data. Of course, both videos and photos are taken into consideration. Both sources are very similar in form. After all, videos are nothing more than a series of images that follow each other in a short time. The word “series” again is key here. A single image may represent a completely different activity to the actual activity.

An outlier in the context of the human movement phase may be a single observation significantly different from the others. In the numerical set, it is an excessive or underestimated acceleration recorded in a given millisecond, and caused, for example, by the oversensitivity of the equipment, the so-called noise, whereas in the image data, it may be an isolated pixel that may owe its different color to the poor quality of the video transmission. This paper focuses on anomalies in a much broader sense, namely, activities within other activities, features in the data that are different from the rest of the dataset. There is then a reasonable probability that they could have been generated by a completely different activity.

For example, imagine a simple case of a person running on a rough road. Most of the time, a well-trained model will recognize the act of running. However, there will be a case that such a person jumps on some bump. The tracking system could then be misled and register this temporary change as a new activity—in this case, jumping.

## 4. Proposed Methods for Detecting Outlier Activity

### 4.1. Nested Binary Classifier

A nested binary classifier (NestBC) was proposed in the article for recognizing human movement activity. The degree of nesting of the classifier is determined by the number of activities that can be recognized.

The individual steps of the algorithm are as follows:

Let $A=\{a_{1},a_{2},\ldots ,a_{k}\}$ denote the set of *k* of activities (for example, {running, walking, lyingdown}, etc.). Prepare a training set $Z_{training}$ and a test set $Z_{test}$, containing labeled classes $C=C_{1},C_{2},\ldots ,C_{k}$ according to the activities included in the analyzed set.

1. Make a copy of the sets $Z_{training}$ and $Z_{test}$ denoting $Z_{training-ki}$, $Z_{test-ki}$, where ki is the copy number (nesting).
2. Choose one activity $a_{i}$ randomly from *A*.
3. In the new sets $Z_{training-ki}$, $Z_{test-ki}$ search for class labels with activity $a_{i}$ and mark classification labels $C_{1}=1$ for activities $a_{i}$ and $C_{0}=0$ for the remaining $a_{i-1}$ activities.
4. Perform a binary classification that recognizes $a_{i}$ activity.
5. In the $Z_{training}$ and $Z_{test}$ sets delete records with the label of the class of activity $a_{i}$.
6. Repeat steps 1–5 until the last activity $a_{k}$.
7. The remaining records, which are unrecognized in subsequent activity nestings, constitute outliers.

The operation of the nested classifier detecting outlier activities can be demonstrated in the following example.

Example No. 1:

Let *A* set contain four activities, such as A={running, lyingdown, standing, sitting}. The $Z_{training}$ training set and the $Z_{test}$ test set contain four classes labeled according to the activities contained in the *A* set. We choose one activity at random, e.g., $a_{2}=lyingdown$. We create new test and training sets, $Z_{training-ki}$ and $Z_{test-ki}$, in which we assign the class label $C_{1}=1$ for activities $a_{2}=\left\{running\right\}$ and $C_{0}=0$ for the remaining $a_{i-1}$ activities, i.e., for activities $a_{1}=\left\{running\right\},a_{3}=\left\{standing\right\},a_{4}=\left\{sitting\right\}$. We carry out binary classification, as a result of which we set records with the recognized activity $a_{2}=\left\{lying\right\}$. We remove all records classified as {lying} from the set and repeat the previous steps for the next activity. In our example, we repeat the steps of the algorithm four times because we have defined four activities. If, in the set, we have already removed the activities recognized in the subsequent steps of the algorithm, and there are still activities for which no label has been assigned, then all the remaining activities are treated as outlier activities.

To sum up, we create (generate) as many classification models as we have defined activities. Each classifier learns to recognize one activity. When learning, it uses the same training sets, but marked differently than the others. The windows (records) representing the activity being recognized are given a label corresponding to its name, and in the next nesting, they do not participate in training. On the other hand, those that obtain a value of 0 are treated as unknown and are classified in the next nesting. The first model that assigns the unrecognized (0 (unknown)) labels to activities passes them on to the next level for reclassification. At each next level of nesting, the model checks whether the observations considered anomalous by its predecessor do not happen to concern its activity, and it also rejected these. The last segments obtained in this way (i.e., by elimination) still appear as outlier activities. The *k*-nearest neighbors method, support vector method, CART decision trees, logistic regression, and naive Bayesian classifier were considered as classification models subject to nesting.

### 4.2. Deep Neural Networks

In our research, we also used a simple deep neural network, for which the number of hidden layers is modifiable. The number of neurons in the hidden layer (responsible for calculations) was set to 100. The number of hidden layers was six. The shape of the network was rectangular. The ReLu activation function (rectified linear activation function, or rectifier function) defined by the Equation (1) was used, where *x* is the input of the neuron.

(1) f(x)=max(0,x)

As far as the hyperparameter *y* is concerned, in each trial, the number of epochs for training and validation was set to 100; however, as was shown in the graphs of the learning curves in Section 6, a much smaller number would have been enough. The proportion of samples fed to the network simultaneously (the so-called batch size) was 40. Additionally, it was assumed that training the network would be stopped if network training did not improve its effects for two consecutive epochs.

Since only one ML algorithm was used in this case, there was no justification or need to submit the results to subsequent majority votes.

## 5. Research Methods

The research concerned the detection of outlying activities in sets containing activities of phases of human movements using the proposed nested classifier and comparing the results with a simple deep neural network. Research experiments that were carried out took into account five classifiers (*k*-NN, naive Bayesian classifier (NB), decision trees (DTs), support vector method (SVM), and logistic regression (LR). Each classifier was tested at a different level of nesting. In subsequent experiments, the operation of classifiers for multiclass classification was checked using deep neural networks.

### 5.1. Algorithm Parameterization

The input parameters of the classifiers for which the best results were obtained are given in Table 1.

Some of the parameters have been left in their default state. The values of others, such as the attributes of neural networks, were selected by a naive trial and error method. The goal was to maximize the number of detected anomalies.

### 5.2. The Measures of Quality of the Classifier

The assessment of the quality of the classifier was made on the basis of known measures, such as accuracy (*ACC*), i.e., the total efficiency of the classifier, and determining the probability of correct classification, i.e., the ratio of correct classifications to all classifications. The second measure of assessment of the classifier quality is precision (*PP*), which indicates the percentage of correct assignments to a given group. We will also determine sensitivity (*SE*), which determines the percentage of observations recognized as belonging to their classes, and specificity (*SP*), which determines the ability of the model to correctly determine the absence of belonging to the considered class. These measures are defined by Formulas (2)–(5), where *TP* is true positive, i.e., correctly assigned to a given class, *FP* is false positive, i.e., incorrectly classified to a given class, *TN* is true negative, i.e., correctly rejected by the algorithm, and *FN* is false negative, i.e., incorrectly rejected by the algorithm. This division refers to binary classification.

(2) $$ACC=\frac{\left(TP+TN\right)}{\left(TP+TN+FP+FN\right)}$$

(3) $$PP=\frac{TP}{TP+FP}$$

(4) $$SE=\frac{TP}{TP+FN}$$

(5) $$SP=\frac{TN}{\left(TN+FP\right)}$$

In the case of multiclass classification, a confusion matrix is determined. It has the form of a square matrix, where one class is marked as positive and the others as negative. This reduces the multiclass problem to multiple binary classification problems. The values from the individual cells of the confusion matrix are used to calculate these classifier evaluation measures. In the case of anomaly detection, an additional criterion was taken into account when assessing the effectiveness of the algorithms used. An important factor was the number of correctly diagnosed anomalous segments. However, due to the disproportion between classes, the number of overmatched outliers was chosen as a negative statistic, i.e., a kind of counterbalance.

### 5.3. Characteristics of Datasets

For the purposes of this research, three datasets containing data representing physical activities in the form of streaming time series were selected. Each stream was defined with a different number of samples and descriptive features. Most of the observations were the so-called raw data, i.e., direct readings from inertial sensors.

Dataset No. 1—Inertia Sensors for Human Activity Recognition (Inertia) [26]—contains data on 11 physical activities split between two sets based on complexity. The first set presents five simple activities, i.e., walking, running, sitting, and going up and going down the stairs. The second set concerns six more complex activities, namely, playing tennis, playing football, cycling, swimming, and performing push-ups. The study used the first set to allow comparison with other sets of similar specifications. In the Inertia dataset, each data sample is defined by the following variables:

- Acceleration (XYZ)—angular acceleration of the device on the axes of the XYZ Cartesian system;
- Gravity (XYZ)—gravitational acceleration;
- Linear acceleration (XYZ)—linear acceleration;
- Gyroscope (XYZ)—spatial orientation;
- The time-since-start—time from the start of sampling, counted in milliseconds;
- Date—date and time the sample was collected.

Dataset No. 2—WISDM [27]—has over 1 million samples. Raw inertial data were collected from 36 users using a mobile phone. In addition to the basic activities, the “standing” activity was also included. The time interval between individual samples is 50 ms. Descriptive variables include:

- User—identity of the test object in the range 1–36;
- Activity—a label that defines the activity to which the sample belongs;
- Timestamp—time since the phone was started in nanoseconds;
- *x*-Acceleration—acceleration on the *x*-axis recorded by the accelerometer in the range <−20,20>, takes into account gravitational acceleration;
- *y*-Acceleration—acceleration on the *y*-axis;
- *z*-Acceleration—acceleration on the *z*-axis.

Dataset No. 3—UCI [28]—has two sets of samples, both in raw and processed form, with just over 10,000 in each one. The time difference between single observations in this case is 20 ms. The first set is described by nine variables relating to all three axes of the Cartesian system, so:

- Body acceleration XYZ;
- Body gyro XYZ—spatial orientation of the body;
- Total acceleration XYZ—i.e., the sum of the tangential acceleration and the normal acceleration perpendicular to it.

In addition, the signals were separated into smaller components (for example, acceleration into body acceleration and gravitational acceleration). For each variable, additional statistical measures were calculated, such as the mean or standard deviation. Thus, the feature vector consists of over 560 attributes. A summary of information on the datasets containing human movement activities used in the research is given in Table 2.

The sets were cleaned and pre-processed. As a result of pre-processing, no missing data were observed in the Inertia collection, but more than 12,000 duplicate rows of data were diagnosed, which were removed so as not to interfere with the learning processes. In the WISDM dataset, 11,740 incorrect rows were detected that were blank, had duplicated columns, or had separators different from the others. About 67,000 duplicated rows were removed. The UCI dataset did not require much intervention. No erroneous or missing values were found, the only intervention was limited to changing the format of the activity labels from numeric values to text.

### 5.4. Segmentation of Samples into Time Windows

Datasets containing activities of human movement phases are data streams; therefore, compression into sets of overlapping time windows of fixed sizes was performed. Due to this, their continuity was preserved. It also allowed us to obtain segments for potential anomalous activities, which was the goal of the described research.

For the Inertia dataset, a split was used in which each window overlapped its neighbors at 50%. Due to the small size of the dataset and the low frequency of the collected samples, each data frame contained only 40 of them. For the WISDM dataset, a window of 144 samples (approx. 7 s) was used, but overlapping at 80%. As a result, the size of each sample increased, and thus, the time needed for learning increased. For the UCI dataset, observations were split between windows overlapping at 50%. Each window contained 128 samples, so the learning time was approximately 2.56 s.

Feature vectors were extended with additional items. For each window of all the descriptive variables on each axis, the following statistics were calculated: mean, median, largest observation, smallest observation, standard deviation, energy, quartile range (difference between the first and third quartile), entropy, correlation with a window with the same index but on a different axis, simple moving average. After the enrichment process, the number of descriptive features in the Inertia dataset increased from 12 to 42, and in the WISDM dataset, the number of descriptive features increased from 3 to 33.

## 6. Experiments and Results

In order to find the best method for detecting outlying segments within human physical activities, a series of classification experiments were carried out. They were aimed at verifying their effectiveness in this task and examining which factor influenced the effectiveness to the greatest extent. Each dataset was divided into two samples—training and validating. Standard proportions were used, i.e., 70% (training set) to 30% (testing set). The selection was carried out randomly so that the segments marked as anomalous did not occur in one sequence, but actually imitated outliers.

### 6.1. Detecting Outlying Activities Using a Nested Binary Classifier

The first experiment used the nested binary classifier discussed in Section 4 at four levels. The tests with the following classifiers were performed sequentially: k-NN, logistic regression, naive Bayesian classifier, CART decision tree, support vector machine (SVM). As described in Section 4, a nested *k*-NN binary classifier at one level learned to detect one activity. Continuing to the last level, it indicated all the other unclassified activities that were searched for as outlying activities.

For the Inertia dataset (Dataset No. 1), four activities were classified: sitting, walking, going_down (going down the stairs), going_up (going up the stairs). The activity that was unknown in the $Z_{training}$ and $Z_{test}$ datasets, i.e., an outlying activity, was the fifth activity, running. Five outliers were detected in this set.

The nested k-NN algorithm (NestBC_k-NN) reached a high match level of 80%. Table 3 lists the evaluation measures for all nested classifiers for each level of nesting. A decrease in accuracy was noted for the two activities, namely, the going_down activity and the going_up activity. This is due to the high similarity between these activities. The nested k-NN classifier left eleven samples as unknown, of which three were true anomalies, and the others belonged to other activities. The two missing samples were mistakenly labeled as going up the stairs. The decision tree, as expected, fared much better, although only three out of five true anomalous segments were detected in the last nesting. The other two actually belonged to the previous activity. One outlier activity was misclassified.

For the nested SVM classifier (NestBC_SVM), at nesting levels III and IV, a decrease in accuracy was observed with the last two activities. In this case, however, it was much more significant. It is likely that the model was overtrained towards the anomalous class due to the large number of descriptive attributes that this algorithm is sensitive to. Among the 23 samples diagnosed as outliers, NestBC_SVM detected all true outlying activities. The remaining 17 belonged to other activities. As many as 11 of these came from the main class of the last nested model.

**Figure 1:**
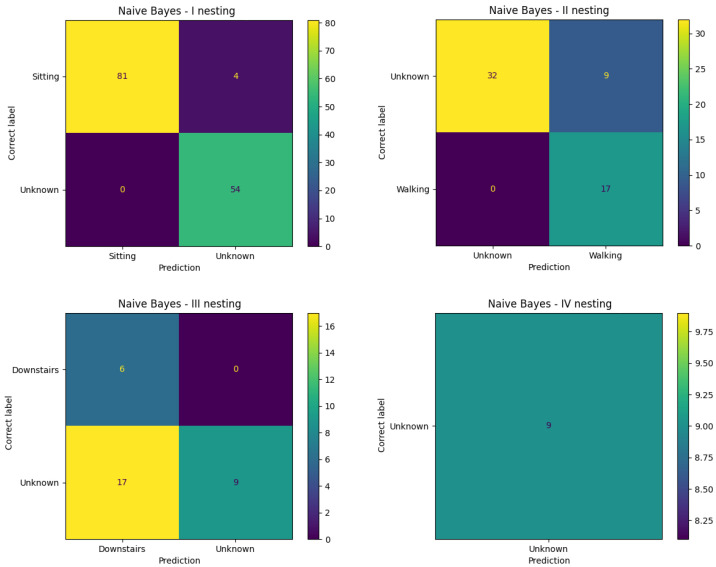
Confusion matrix for a nested naive Bayesian classifier for the Inertia dataset.

In the case of the nested naive Bayesian classifier (NestBC_NB), so-called undertraining of the model was observed. The confusion matrix is illustrated in Figure 1.

At level I, the algorithm coped very well with recognizing the numerous sitting classes. However, as soon as more dynamic activities appeared, its effectiveness began to decrease significantly, and the algorithm had great difficulty in distinguishing them. The reason for this is probably the small number of samples present during training. However, it should be emphasized that all five anomalous segments were found by the final nesting. Interestingly, the remaining four samples belonged to walking activity, and thus, these were the only ones that were misclassified in the first level. The algorithm correctly detected all fragments of going down the stairs, but also classified all going up segments as going down. This reinforces the assumption that the model was not able to distinguish between the two activities at first.

**Figure 2:**
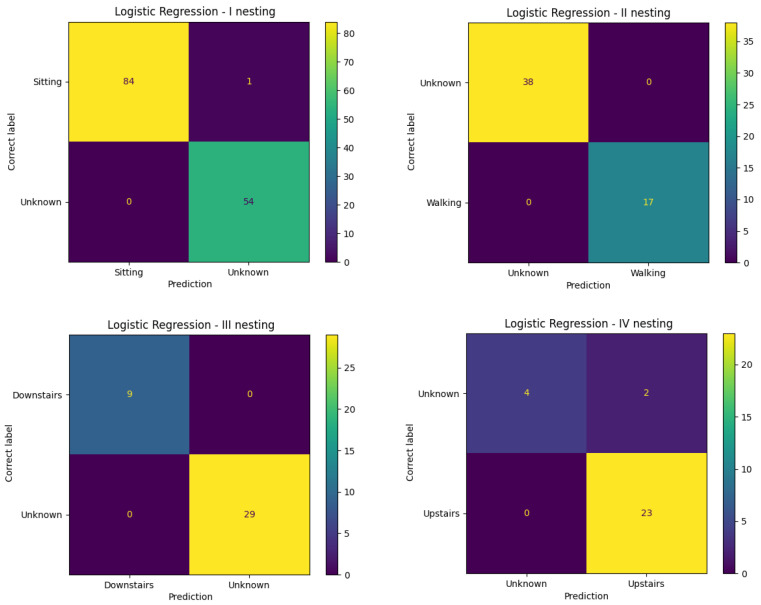
Confusion matrix for the nested logistic regression classifier for the Inertia dataset.

In the case of the nested logistic regression model, based on the confusion matrix illustrated in Figure 2, it is easy to see a small number of misclassified samples at each level. The model achieved the best results for all calculated measures evaluating the performance of the classifier given in Table 3. Only three out of four observations were actually anomalous. The other two were classified as “walking”. The anomalous class was dominated by the more numerous going up the stairs class.

For the second dataset (WISDM) (Dataset No. 2), the following four activities were considered: sitting, walking, and going down and going up the stairs. Standing and running were outlier activities. The number of actual outlier activities was 5219. As before, experiments were performed for each classifier. The evaluation measures of the nested classifiers for the WISDM dataset are given in Table 4.

With each successive nesting, the accuracy of the model slightly decreased, but still remained at a high level. In the last phase, 5287 anomalous activities were received, of which 5158 actually belonged to this classification. The remaining 129 were misclassified activities.

The nested decision tree algorithm performed slightly worse than the nested kNN model. Also, this time there is a significant improvement compared to the previous dataset. A smaller number of incorrectly classified static activity segments was obtained, as compared to the Inertia dataset. However, the number of false positive and false negative observations in dynamic activities increased. In none of the nestings did the accuracy fall below 94%. Precision and sensitivity were not less than 81%. Specificity was also very high. The exact values of all assessment measures are given in Table 4. In total, 5105 true outlier cases were found, while 663 were overmatched.

For the WISDM dataset, the nested support vector machine on the first level selected the segments of sitting activity without major problems. With the emergence of outlier activity, the effectiveness decreased. At levels III and IV, the anomalous class was clearly dominant. In the end, 5173 anomalies were correctly classified, but a huge percentage, comprising as many as 2829 samples, were matched incorrectly by the nested SVM classifier. For the nested naive Bayes classifier, it was noticed that the model was guided by the predominance of the number of representatives of a given class (again, anomalous). A total of 4221 valid anomalous samples were detected, while the missing 998 were probably lost in the first two nestings. The nested logistic regression model for the WISDM dataset performed very well. In none of the nests was accuracy over 88%. It was also noticed that the nested logistic regression model coped worse with activities that require movement. The numbers of incorrectly classified samples were the largest at the second, third, and fourth level of the model. In the end, 4942 real outliers were detected, while the remaining 2309 belonged to other classes.

For the third analyzed dataset of UCI (Dataset No. 3) human movement activities, the correct activities included sitting, walking, and going down and going up the stairs. Standing and lying down were outlier activities. The number of actual outlier activities was 1069. The nested binary k-nearest neighbors algorithm coped very well with this last dataset. It is worth noting that even distinguishing very similar activities on the last two levels was not a major problem. Very small numbers of incorrectly diagnosed segments were obtained (I (nesting)—37, II—14, III—16, IV—42). Accuracy, precision, sensitivity, and specificity ranged from 88% to 99%. The measures of nested classifier evaluation at particular levels are given in Table 5. Accuracy did not drop below 96% at any level of nesting, even with the dominance of the anomalous class. In the end, 1032 of the real outliers were correctly detected, and 130 samples were overmatched.

For the nested CART decision tree, 998 out of 1069 anomalous segments were classified, and 367 observations were overmatched. According to Table 5, the lowest accuracy value, i.e., about 91%, was obtained in the last nesting. The nested binary support vector machine turned out to be much more efficient than in the case of the WISDM dataset. The nested SVM model correctly diagnosed 1041 anomalous segments, whereas 131 fragments were incorrectly assigned with this class. Accuracy never fell below 96%. The nested naive Bayes classifier for UCI dataset behaved in a rather surprising way. For the Inertia dataset and WISDM dataset, the accuracy decreased with subsequent nestings. In this case, the first level was characterized by the lowest percentage of correctly matched labels, while the last level was characterized by the highest. Nevertheless, it was noted that there was overmatching, resulting in the detection of only 78 segments. Only 18 incorrect segments were observed. For the nested logistic regression model, 1056 anomalies were detected. Only 134 of the finally obtained outliers were incorrect. The evaluation measures of the nested logistic regression model (Table 5) confirm its high quality for the detection of outlier activities. Accuracy did not fall below 97% at any level. Precision had the biggest drop at the second level (96%), contrary to sensitivity, which then reached the highest value. No major differences were observed between individual nestings.

In conclusion, the nested classifier works correctly, and can be used to detect anomalous activities of human movement phases. The disadvantage, however, is that it can generate many additional outliers at the same time, which is certainly not a desirable phenomenon.

### 6.2. Experiment II—Detecting Outlier Activities Using a Deep Neural Network

The aim of the second experiment was to test the efficiency of outlier activity detection using deep neural networks. The deep learning method described in Section 4 was used for testing. The test was performed several times with modifying the network parameters. The best results are presented below. Again, due to the nature of the task, there was no need to change the anomalous class. Because only one machine learning method was used, no additional voting was performed.

#### 6.2.1. Dataset No. 1—Inertia

**Figure 3:**
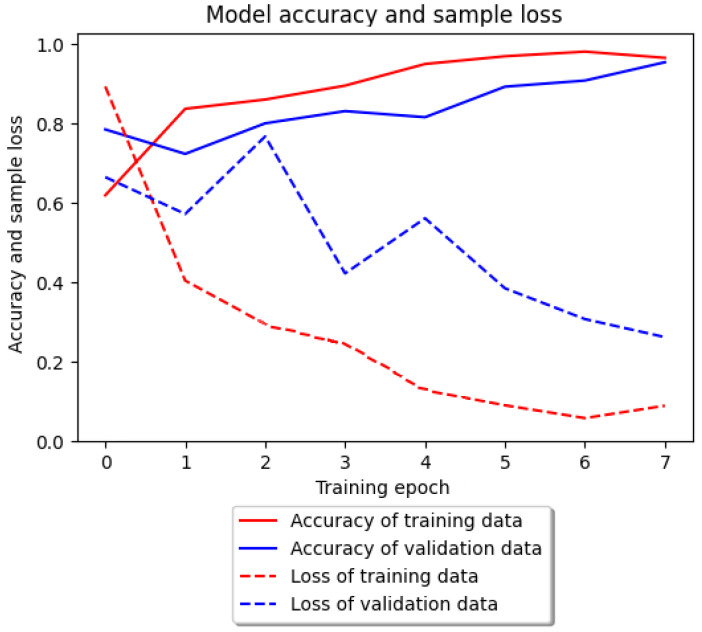
Accuracy of the classification model and loss of samples for the Inertia dataset for deep learning.

The accuracy of the classification and the number of missing samples in relation to the number of epochs for both of these tests are shown in Figure 3.

**Figure 4:**
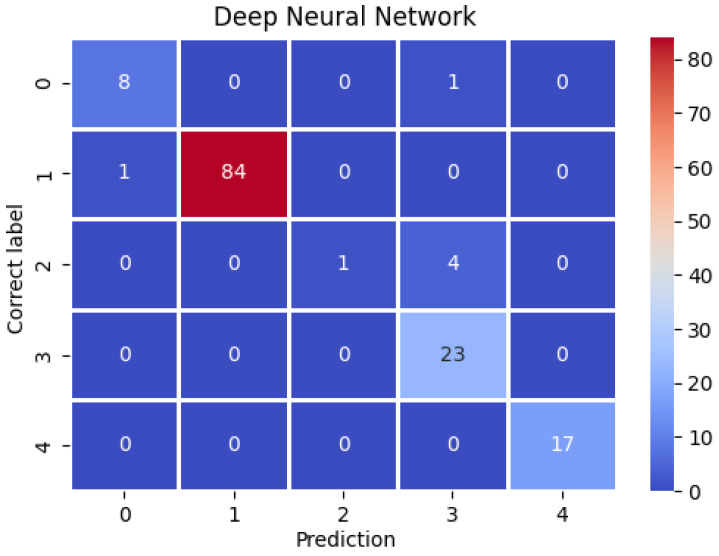
Confusion matrix of a deep neural network for the Inertia dataset.

It should be noted that for the training sample, the greatest increase in accuracy took place during the first epoch. Then, it increased through successive iterations until it reached its peak in epoch 6, after which it began to gradually decrease. In the case of the test sample, the initial decline turned into a gradual increase, peaking at the end of epoch 7. Only the anomalous class segments and a single stair climbing sample were misdiagnosed. In the other cases, no deviations were observed. The confusion matrix for the Inertia dataset is shown in Figure 4. The obtained precision, sensitivity, and specificity measures obtained for the Inertia dataset using deep neural networks are given in Table 6. Accuracy (ACC) was ACC=98%. This is the highest percentage of correctly classified segments obtained during the experiments so far. We managed to detect three out of five real anomalies.

#### 6.2.2. Dataset No. 2—WISDM

**Figure 5:**
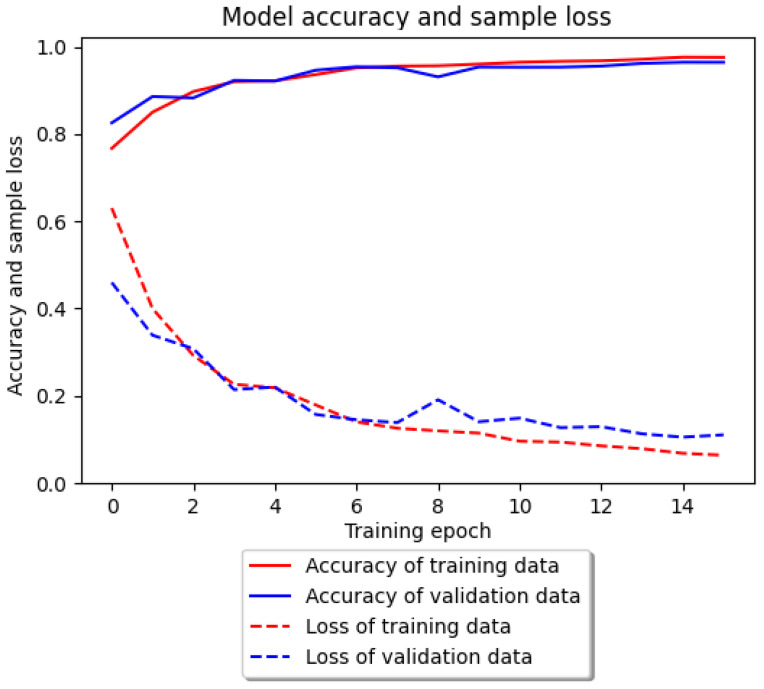
Accuracy of the classification model and loss of samples for the WISDM dataset for deep learning.

The accuracy of the classification model and loss of samples for the WISDM dataset are shown in Figure 5.

Based on Figure 5, it can be seen that during the training of the WISDM dataset, the neural network model was gradually improving its accuracy. This was also the case for the test sample with a small outlier in epoch 8. The whole process needed about 15–16 epochs to fully stabilize the results. The second trial, using a much larger dataset, was also characterized by a high-quality model. The percentage of correctly classified samples was approximately 96.40%. In total, 5162 out of 5219 samples were detected. Variations in the classifier evaluation measures were found for the going_up and going_down classes. The lowest precision value was observed for the going_up class at 85%, and the lowest sensitivity for the going_down class at 93%.

#### 6.2.3. Dataset No. 3—UCI

**Figure 6:**
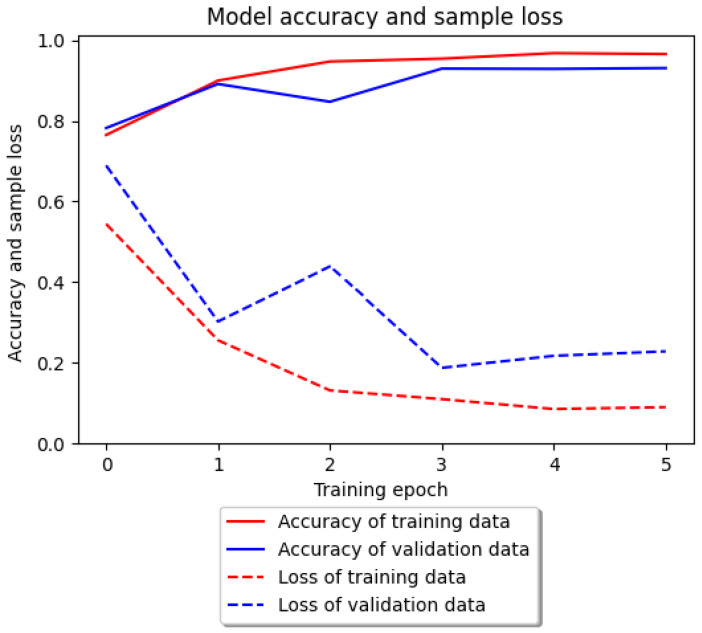
Accuracy of the classification model and sample loss for the UCI dataset for deep learning.

For the UCI dataset, the course of both the training and the testing was very similar to the previously described results. The greatest increase in accuracy took place in the first epoch, after which it stabilized until it reached a state of complete stagnation. As with the previous dataset, a slight decrease was observed in the test sample after the end of the second epoch. However, the decrease in the number of samples was much smaller than in previous experiments. The accuracy of the classification and the number of missing samples in relation to the number of epochs for the UCI dataset are shown in Figure 6. In this case, the model predicted labels for 91.65% of all items. Overall, 908 out of 1069 unique segments were identified. The result, although the lowest so far, is still satisfactory. The obtained measures of deep neural network classification evaluation for the UCI dataset are given in Table 7. The lowest precision was PP=82%. The lowest sensitivity was SE=81%.

Summarizing the last experiment, the deep learning of neural network seems to be an extremely effective technique for detecting unique segments of activity. With a relatively quick learning and validation time, you can obtain precise indications while not worrying about a high percentage of overmatched anomalies. Its true strength lies precisely in the unique learning process, because it does not require specially processed datasets. The first tests carried out on networks using raw data (indications of accelerometer sensors) produced results slightly worse than those obtained from datasets enriched with additional attributes. This shows that, for this ML algorithm, the most important factor is the number of samples available for training, not their extensive description.

## 7. Discussion

Summing up the conducted research and experiments, for clarity and an easier summary, a list of the number of correctly classified anomalous segments for the nested binary classifier and the deep neural network (GNN) was generated, and is given in Table 8, where NestBC_k-NN means the nested binary classifier *k*-nearest neighbor, NestBC_DT_CART is the nested decision tree classifier CART, NestBC_SVM is the nested support vector classifier, NestBC_NB is the nested naive Bayes classifier, and NestBC_RL is the nested logistic regression classifier. Due to the significant differences in the sizes of the datasets, to enable a better comparison of the models, the percentages of detected outliers included in the test datasets are also given.

In the first Inertia dataset, the nested binary SVM models and the naive Bayes classifier were characterized by the highest efficiency. Most algorithms were only able to detect 60% anomalies. However, using them in a multiclass task after stabilizing by majority vote improved accuracy. Of course, it should be borne in mind that this dataset is very limited in terms of the number of samples, and although the percentage differences may seem significant, they are in fact only single samples.

For the second dataset, almost all models passed the 90% accuracy threshold. The first place in this respect was taken by the nested support vector machine algorithm. The second place was taken by the deep neural network. The nested k-nearest neighbors algorithm fared slightly worse. The differences this time were single or even decimal percentage points. An outlier to this rule, however, was the nested binary Bayes classifier, which lost a large part of the samples during the first iteration. In the last, most attribute-rich UCI dataset, the largest number of real anomalous segments was detected by the nested logistic regression algorithm.

Most models passed the 90% threshold. Once again, the nested binary model using the Bayesian algorithm was not able to cope with the extensive dataset. It can therefore be concluded that the classifier models faced with a complex binary classification task are capable of detecting outlying fragments of activity at a similar level as neural networks. A thorough analysis of the confusion matrix, however, led to the introduction of one more criterion when evaluating the effectiveness of these models. It was noticed that each of the trials generated a certain number of mistakenly overmatched samples. Table 9 lists the number of samples incorrectly flagged as anomalous.

Of course, it is difficult to obtain perfect models, but in the task of detecting outliers, generating any additional ones is an undesirable phenomenon. It should, if possible, be removed or at least limited. After studying this table, there is no doubt that binary nested models, while being as accurate as the other models presented in this paper, also create a larger number of additional outliers.

## 8. Conclusions

To sum up, this work aimed to investigate which of the popular machine learning algorithms can be used in detecting anomalies in the phases of human movement. Studies were performed using nested classifiers and a deep neural network. The methods were subjected to different methods of validation under different conditions. The research was carried out on three numeric datasets with different characteristics.

The comparison of classifiers showed that almost all of the used algorithms coped with the detection very well, even when the training sample was limited. The best results were achieved using NestBC SVM classifiers and deep neural networks. The method of nested binary classifiers can be considered an effective way of recognizing outlier patterns. In most cases, its results did not differ significantly from other methods. However, the proposed method also generates a lot of false predictions, which is certainly not a desirable phenomenon. Even with its high accuracy, the act of creating additional anomalies seems to disqualify it from potentially taking part in such tasks. An outlier to this rule may be the nested k-nearest neighbors algorithm.

It is important to mention that, for example, the kNN algorithm, being trained on the WISDM dataset, did not exceed 65% accuracy in the test sample, while only deep neural networks managed to achieve at least 90%. This shows that classification models require not only extensive but also attribute-rich datasets to be high-performing. This also illustrates the advantage of deep neural networks, which do not seem to be affected by this dilemma. It becomes even more important when taking into account the abovementioned problem of the low availability of processed datasets regarding human physical activities. In future research, we will test different types of deep learning methods and compare them with a nested binary classifier.

## Figures and Tables

**Table 1:** Algorithm parameters.

Algorithm Name	Parameters
k-NN	Number of neighbors—7
DT-CART	Depth—no limits
SVM	Default
NB	None
LR	Maximum iteration—500
Deep Neural Networks	Number of epochs—100
	Number of hidden layers—6
	Number of neurons in the hidden layer—100

**Table 2:** Summary of the most important information about selected datasets.

Name of the Dataset	Number of Samples	Number of Classes	Number of Attributes
Inertia	23,346	5	14
WISDM	1,098,207	6	6
UCI	10,299	6	561

**Table 3:** Evaluation measures (%) of nested classifiers at each level for the Inertia dataset, where k-NN means k-smallest neighbors; DT_CART—CART decision trees; SVM—support vector machine; NB—naive Bayesian classifier; LR—logistic regression.

Nested Classifier	Level of Nesting	Accuracy	Precision	Sensitivity	Specificity
NestBC_kNN	I	98.56	100.00	97.64	100.00
	II	100.00	100.00	100.00	100.00
	III	89.74	77.77	77.77	93.33
	IV	80.00	89.47	80.95	77.78
NestBC_DT_CART	I	99.28	98.84	100.00	98.15
	II	100.00	100.00	100.00	100.00
	III	97.22	90.00	100.00	96.30
	IV	88.46	95.23	90.91	75.00
NestBC_SVM	I	98.56	100.00	97.65	100.00
	II	100.00	100.00	100.00	100.00
	III	79.49	57.14	44.44	90.00
	IV	65.63	100.00	45.00	100.00
NestBC_NB	I	97.12	100.00	95.29	100.00
	II	84.48	65.38	100.00	78.05
	III	46.87	26.08	100.00	100.00
	IV	100.00	0.00	0.00	0.00
NEstBC_LR	I	99.28	100.00	98.82	98.82
	II	100.00	100.00	100.00	100.00
	III	100.00	100.00	100.00	100.00
	IV	93.10	92.00	100.00	100.00

**Table 4:** Nested classifier evaluation measures at individual levels of the WISDM dataset (%).

Nested Classifier	Level Nesting	Accuracy	Precision	Sensitivity	Specify
NestBC_k-NN	I	99.68	97.97	96.36	99.88
	II	99.09	99.22	98.62	99.43
	III	98.11	94.58	94.32	98.90
	IV	97.67	93.35	97.29	97.80
NestBC_DT-CART	I	99.85	98.82	98.36	99.93
	II	96.87	96.31	96.27	97.32
	III	94.38	83.14	81.61	96.83
	IV	94.67	91.35	82.52	97.92
NestBC_SVM	I	99.42	99.22	90.26	99.96
	II	89.06	84.19	91.01	87.64
	III	88.36	0.00	0.00	100.00
	IV	84.22	96.00	1.86	99.99
NestBC_NB	I	95.81	57.26	94.36	95.90
	II	73.67	63.45	94.24	57.61
	III	98.93	0.00	0.00	99.96
	IV	94.96	1.85	0.51	98.87
NestBC_LR	I	99.62	98.06	95.07	99.89
	II	86.51	84.42	83.38	88.80
	III	88.07	62.39	37.39	96.33
	IV	88.30	65.86	34.87	97.04

**Table 5:** Nested classifier evaluation measures at individual levels for the UCI dataset (%).

Nested Classifier	Level Nesting	Accuracy	Precision	Sensitivity	Specify
NestBC_k-NN	I	95.42	91.39	80.04	98.99
	II	96.62	87.16	97.17	96.49
	III	96.53	95.38	86.39	98.99
	IV	96.23	90.78	95.61	96.46
NestBC_DT-CART	I	93.69	84.12	76.58	97.11
	II	94.32	86.57	84.48	96.76
	III	93.55	91.32	75.12	98.20
	IV	90.61	87.70	70.03	96.97
NestBC_SVM	I	97.39	94.04	90.02	98.86
	II	98.67	95.84	97.58	98.94
	III	97.67	99.73	88.97	99.94
	IV	96.64	96.79	91.34	98.78
NestBC_NB	I	65.80	32.35	96.53	59.65
	II	51.75	40.88	98.99	27.99
	III	89.68	70.18	76.92	92.58
	IV	92.86	88.28	99.12	86.36
NestBC_LR	I	97.56	97.08	87.98	99.47
	II	99.08	96.28	99.19	99.05
	III	98.04	99.48	91.11	99.87
	IV	97.55	99.52	91.67	99.83

**Table 6:** Classifier evaluation measures of precision (PP, %), sensitivity (SE, %), and specificity (SP, %) obtained for the Inertia dataset using a deep neural network.

Class	PP	SE	SP
going_down (0)	90.00	100.00	99.23
sitting (1)	100.00	99.00	100.00
outlier (2)	100.00	60.00	100.00
going_up (3)	92.00	100.00	98.28
walking (4)	100.00	100.00	100.00

**Table 7:** Classifier evaluation measures of precision (PP, %), sensitivity (SE, %), and specificity (SP, %) obtained for UCI dataset using a deep neural network.

Class	PP	SE	SP
going_down (0)	92.00	89.00	98.73
lying (1)	100.00	95.00	100.00
sitting (2)	95.00	81.00	99.10
going_up (3)	87.00	97.00	97.29
outlier (4)	82.00	96.00	95.32
walking (5)	97.00	91.00	99.51

**Table 8:** The number of correctly detected outlier activities (*N*) and obtained accuracies (%) of outlier detection for nested binary classifiers and deep neural networks in each analyzed dataset.

Dataset	Inertia		WISDM		UCI	
	*N*	Accuracy (%)	*N*	Accuracy (%)	*N*	Accuracy (%)
NestBC k-NN	3	60.00	5158	98.83	1032	96.54
NestBC DT_CART	3	60.00	5105	97.82	998	93.36
NestBC SVM	5	100.00	5173	99.12	1041	97.38
NestBC NB	5	100.00	4221	80.88	78	7.29
NestBC RL	3	60.00	4942	94.69	1056	98.78
GNN	3	60.00	5162	98.91	1021	95.51

**Table 9:** Number of overdetected anomalies for individual algorithms (NestBC_k-NN—nested k-NN classifier, NestBC_DT_CART—nested CART decision tree, NestBC_SVM—nested support vector machine, NestBC_NB—nested naive classifier Bayes, NestBC_RL—nested linear regression classifier, GSN—deep neural network).

Dataset	NestBC k-NN	NestBC DT_CART	NestBC SVM	NestBC NB	NestBC RL	GSN GSN
Inertia	8	2	17	4	1	0
WISDM	129	663	2829	628	2309	48
UCI	130	367	131	18	134	113

## Data Availability

Not applicable.
